# Higher Mobility Scores in Patients with Cystic Fibrosis Are Associated with Better Lung Function

**DOI:** 10.1155/2015/423219

**Published:** 2015-02-18

**Authors:** Aneesha Thobani, Jessica A. Alvarez, Shaina Blair, Kaila Jackson, Eric R. Gottlieb, Seth Walker, Vin Tangpricha

**Affiliations:** ^1^Division of Endocrinology, Metabolism and Lipids, Department of Medicine, Emory University School of Medicine, Atlanta, GA 30322, USA; ^2^Nutrition Health Sciences Program, Graduate Division of Biological and Biomedical Sciences, Emory University, Atlanta, GA 30322, USA; ^3^Emory University Cystic Fibrosis Center, Atlanta, GA 30322, USA; ^4^The University of Maryland School of Medicine, Baltimore, MD 21201, USA; ^5^Division of Pulmonary, Allergy and Critical Care Medicine, Department of Medicine, Emory University School of Medicine, Atlanta, GA 30322, USA; ^6^Atlanta VA Medical Center, Decatur, GA 30022, USA

## Abstract

*Objective.* The purpose of this study was to determine whether mobility and physical activity were associated with lung function in adults with cystic fibrosis (CF). *Design.* This was a prospective cohort observational study in an urban, academic, specialized care center. Participants were ambulatory, nonhospitalized adults with CF. *Main Outcome Measures.* Mobility was assessed monthly by the Life-Space Assessment (LSA) questionnaire and quarterly by pedometer. Lung function was assessed by spirometry. *Results.* Twenty-seven subjects participated. Subjects recorded mean pedometer steps of 20,213 ± 11,331 over three days and FEV_1_% predicted of 77.48% ± 22.60% over one year. The LSA score at enrollment was correlated with initial pedometer steps (*r* = 0.42 and *P* = 0.03), and mean LSA score over one year was correlated with mean number of steps (*r* = 0.51 and *P* = 0.007). LSA mobility and pedometer scores were correlated with FEV_1_% predicted at enrollment and throughout the study. *Conclusions.* Mobility and physical activity measured by LSA questionnaire and pedometer are positively associated with lung function in adults with CF. This study confirms the importance of mobility and physical activity and supports the utility of a simple office-based questionnaire as a measure of mobility in adults with CF.

## 1. Introduction

Cystic fibrosis (CF) is a hereditary, chronic respiratory illness in which patients suffer from recurrent bouts of infection causing frequent hospitalization [1–3]. Patients with CF experience a chronic decline in lung function, and those with end-stage lung disease may become candidates for lung transplant. However, patients with higher levels of physical activity may have lower rates of decline in lung function, improved airway clearance, better muscle function, and enhanced quality of life [4, 5].

*Review of the Literature.* The importance of physical activity in patients with CF is well supported. Physical activity may include moderate to vigorous exercise associated with structured training and athletics, as well as day-to-date activity, termed habitual physical activity (HPA) [4–6]. Physical activity is associated with increased cardiovascular endurance, muscle strength, mucus clearance, and quality of life [4]. Ideally, patients should perform a combination of aerobic and strength training since aerobic training improves peak aerobic capacity, activity levels, and quality of life, while resistance training improves weight gain, FEV_1_% predicted, and strength [4]. Strength training and aerobics both increased work capacity in patients with CF and demonstrated an increase in FEV_1_% predicted [6, 7]. An increased level of HPA, independent of structured exercise, is also associated with better health outcomes in patients with CF, including improved aerobic capacity and lower rates of respiratory decline [8]. There are significant barriers to physical activity, including HPA, such as muscle defects, poor health, poor nutrition, and the constraints of intensive therapy and frequent hospitalization. Patients may also self-limit their HPA due to the perception of poor health and vulnerability to infection and other adverse events [4].

A reliable patient-reported measure of HPA, independent of overall quality of life, could provide an early indication of patients who are at risk for CF-related morbidity. Physical activity questionnaires contain limited reliability and validity, as they are prone to recall bias [9]. There exist such questionnaires as the Cystic Fibrosis Questionnaire-Revised (CFQR) [10], CF Respiratory Diary [11], Cystic Fibrosis Quality of Life (CFQoL) [12], and Questions on Life Satisfaction [13], all of which are directed towards patient-reported outcomes but only target disease-specific quality of life measures, while not targeting physical activity specifically. Aside from questionnaires, there are limited tools for measuring physical activity level specifically in patients with CF. Pedometers have been used previously and are feasible for use in the CF population; steps measured with a pedometer correlate with changes in health status and can be used as an outcome measure in CF [14]. A questionnaire that produces a score consistent with pedometer steps could minimize the time and expense of assessing physical activity in patients with CF and prove useful in clinical practice.

The Life-Space Assessment (LSA) is a validated tool used to measure mobility patterns in geriatric patients [15, 16]. The LSA measures how far and how often a person travels from his or her dwelling space and the level of independence that he or she exhibits [15]. In these previous studies, the LSA was shown to be associated with physical performance, cognitive abilities, mental health, and rate of recovery following surgical and nonsurgical hospitalization [15, 17–21]. Because of the accelerated decline in function and reduced life expectancy in CF, we previously hypothesized that this instrument designed to assess changes in functional capacity might be applicable to this patient population as well. In a retrospective study, we showed that Life-Space scores were associated with FEV_1_% predicted and negatively correlated with rates of hospitalization in adults with CF [16]. Thus, the LSA may be a useful tool to assess health status in CF. It was not known, however, whether Life-Space scores correlate with mobility in the CF population and whether they are predictive of future changes in lung function.

*Purpose.* The purpose of this study was to determine if mobility as measured by pedometers and the LSA questionnaire was associated with improved lung function in patients with CF. We hypothesized that patients who have higher mobility by both measures would have better lung function as measured by FEV_1_% predicted.

## 2. Materials and Methods

### 2.1. Participants and Protocol

The study was approved by the Emory Institutional Review Board. All participants provided written informed consent for participation in this study. Participants were recruited during outpatient clinic visits at the Emory University Adult Cystic Fibrosis Center in Atlanta, Georgia, between March 2011 and May 2012. Inclusion criteria for this study were a CF diagnosis, being clinically stable, and age ≥18 years. Participants were excluded if their clinic visit indicated need for hospitalization and/or acute exacerbation. Upon enrollment, subjects completed the LSA. Subjects completed the LSA monthly, either during clinic visits or by phone interview conducted by study investigators. Demographic and clinical characteristics were extracted from subjects' medical records. Thirty-five participants were recruited for the study, out of which 27 completed the one-year follow-up visit.

### 2.2. Assessment of Physical Activity

Subjects were provided with a pedometer (New Lifestyles DIGI-WALKER SW-200, New-Lifestyles Inc., Lee's Summit, Montana) that they were asked to wear for three consecutive days quarterly for one year. Three days has previously been reported to provide a sufficient estimate of weekly pedometer-assessed physical activity [22]. Self-reported pedometer readings were collected by phone quarterly throughout the one-year study period. We collected information quarterly to account for potential seasonal variation in physical activity.

### 2.3. Life-Space Assessment

The LSA score is a measure of the frequency and independence of travel to different areas extending outward from one's dwelling space during the previous four weeks [15, 17–21]. This instrument was initially designed for the geriatric population, but we have shown that it may be appropriate for use in the CF population as well [16]. The LSA questionnaire assesses the frequency and level of independence that subjects exhibit in traveling to rooms in their homes other than the one in which they sleep (level 1); areas outside their homes in their yards or driveways (level 2); places in their neighborhoods other than their own yards or driveways (level 3); places outside of their neighborhoods but still within their towns (level 4); and places outside of their towns (level 5). For each level a subject reached, he or she was asked to report the frequency of attaining that specific level in the past four weeks (daily (score = 4), 4–6 times per week (3), 1–3 times per week (2), or less than once a week (1)). Subjects were also asked to report their levels of independence based on whether they required personal assistance (1) or equipment (1.5) or exhibited complete independence (2). The scores for each level were summed to calculate a total with a possible maximum score of 120. A higher LSA score is indicative of a larger “life-space” or zone of living for a subject. See Peel et al. 2005 [15] for an in-depth description of the LSA.

### 2.4. Statistical Methods

Descriptive statistics were compiled. The average number of steps by pedometer and average LSA score were recorded at time of enrollment and over the course of the year. Pearson correlation analyses were used to assess the relationship between LSA scores and reported numbers of pedometer steps at baseline, as well as average one-year scores and steps, respectively. All statistical analyses were performed using the JMP Pro 10 software package (SAS Institute Inc., Cary, NC) and assumed a statistical significance value of *P* < 0.05.

## 3. Results

### 3.1. Study Subjects

A total of 35 subjects consented to participation in this study. Twenty-seven subjects completed the one-year follow-up study visit. Dropouts were primarily due to inconvenience of monthly phone calls or inability to be contacted by phone. The study demographics for the 27 participants are presented in Table 1. Complete pedometer data were available for 24 subjects.

### 3.2. Life-Space Score and Pedometer Readings

Subjects reported a mean (± SD) of 19,452 ± 10,118 steps over three days at enrollment and a mean of 20,213 ± 11,331 steps over three days recorded quarterly throughout the year. Subjects reported a mean (± SD) LSA score of 90.39 ± 22.98 out of 120 at baseline and a mean LSA score of 91.94 ± 20.64 recorded quarterly throughout the year (Table 1).

**Figure 1:**
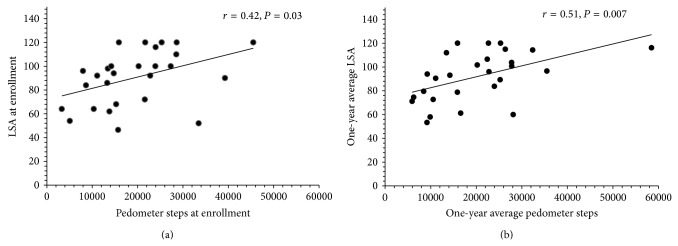
Relationship between Life-Space Assessment score and steps recorded by pedometer at enrollment (a) and over 1 year (b). The Life-Space Assessment score was positively associated with physical activity as assessed by pedometers in adult subjects with cystic fibrosis at enrollment and after 1 year of follow-up.

The mean LSA score at enrollment was positively correlated with number of pedometer steps at enrollment (*r* = 0.42 and *P* = 0.03). The mean LSA score over one year was positively correlated with mean number of pedometer steps recorded during the year (*r* = 0.51 and *P* = 0.007), as shown in Figure 1.

### 3.3. Life-Space Score and Lung Function

Participants had a mean (± SD) FEV_1_% predicted of 77.48 ± 22.60%. Both enrollment and one-year average LSA were associated with lung function as measured by FEV_1_% predicted (*r* = 0.62 and 0.67, resp.; *P* < 0.001 for both).

### 3.4. Pedometer Steps and Lung Function

Both enrollment and one-year average pedometer steps were associated with FEV_1_% predicted (*r* = 0.39 and 0.40, resp.; *P* = 0.04 for both).

## 4. Discussion

In this study we examined the relationship between the LSA score and mobility assessed with pedometer step counts and the correlation of both measures with lung function. We found a significant positive correlation between the LSA score and number of pedometer steps, both at enrollment and throughout the year. Subjects with higher LSA scores reported a greater number of steps. This study provides preliminary validation of the LSA as an instrument to assess mobility and shows that greater mobility is associated with better lung function in patients with CF.

Physical activity is associated with maintenance or improvement of health status in patients with CF [4–7]. Questionnaires have been used to assess the health status of patients with CF [23], including the Short-Form Health Survey (SF-36) [24], the Sickness Impact Profile (SIP) [25], and the Nottingham Health Profile (NHP) [26], but neither of these directly measures physical activity, and they are relatively time-consuming for physicians and other clinicians to administer. The Cystic Fibrosis Quality of Life (CFQoL) has been used to address issues specific to patients with CF [12], and of the 52 questions, some are directed towards the subjects' mobility. However, the CFQoL aims to assess the psychosocial implications of the disease and is not a validated tool for mobility and/or physical activity levels. Pedometers have been validated for the measurement of physical activity [14], but these are not readily available in most physicians' offices, and data collection requires frequent follow-up and may be a burden to patients and clinicians.

The LSA is short and concise and evaluates a subject's mobility as measured in five zones extending outwards from the closest dwelling space. It has been validated as a predictor of health in the geriatric population, and studies have shown that subjects with a higher LSA score are more mobile [15, 17–21]. LSA score is associated with standard indicators of health in patients with CF [16]. This prospective study confirms these findings and validates them as a measure of mobility in the same population.

In our study, LSA score was positively associated with the subjects' lung function as measured by FEV_1_% predicted. Pedometer step counts were also associated with better lung function. Numerous reasons potentially explain our interrelationships between LSA score, pedometer step counts, and lung function. Greater step count could also reflect greater mobility, which may, in turn, indicate greater access to medical care (i.e., clinic visits), better treatment adherence, greater quality of life, and/or greater functioning in general, all of which would influence lung function [27, 28]. However, studies conducted in patients with COPD and obstructive lung disease also show that a lower number of steps is associated with severe physical inactivity and increases the risk for disability [29, 30]. We hypothesize that the same would be true in CF, which is also an obstructive lung disease characterized by progressive disability. In patients with CF, LSA score and step count may also reflect habitual physical activity, which was shown in a longitudinal study to be associated with lower rates of respiratory decline [8]. Beneficial effects of physical activity specific to CF may include serving as an adjunct to physiotherapy by agitating the characteristic mucus, strengthening the chest wall musculature, and increasing physical work capacity [4, 7]. Aerobic exercise as well as habitual physical activity may also strengthen the chest wall musculature, which could improve pulmonary function. Strength training has been shown to increase physical work capacity, which may contribute to pulmonary rehabilitation as well [7].

A causal directionality cannot be determined with these studies. It is possible that impaired lung function precludes mobility and physical activity. Expression of defective CFTR in skeletal muscle may directly cause a reduction in work capacity and make patients with CF vulnerable to deconditioning [31]. For this reason as well, it is important to monitor physical activity in patients with CF. Some of the association may be explained simply by the fact that an individual with better lung function may be more capable of carrying out an active lifestyle and would therefore have a higher Life-Space score. Similarly, there are other psychosocial factors including perceptions of disease burden and constraints of treatment that may affect physical activity in patients with more severe disease [32]. Conversely, physical activity has been shown to affect perception of disease burden, emotional functioning, and various other subsets of quality of life scales. This in turn may improve treatment adherence. It may also increase a patient's motivation to exercise even more through positive feedback [27, 33]. Habitual physical activity may be an important mediator of quality of life because it is more accommodating to include the activities that a patient enjoys and is more sustainable than a formalized training program.

Our data suggest that LSA is an efficient and effective instrument for evaluating the health status of a patient with CF. It provides information that can be shared among the multiple clinicians that participate in the care of a patient with CF, including physicians, nurses, dietitians, respiratory therapists, and social workers. It is taken for granted that all forms of physical activity are beneficial for patients with CF, to the extent that it was considered unethical to perform a study in which some patients would be randomized not to receive exercise training [7]. If a patient visiting a CF clinic has a low Life-Space score, the clinician should investigate whether the patient is in poor health or otherwise should suggest an intervention to increase physical activity in daily life.

One limitation of this study was the small sample size. Additionally, this instrument is designed to assess habitual mobility, or “life-space,” rather than vigorous exercise or habitual physical activity, which the LSA does not capture. Thus it would not be used alone to fully measure physical activity or exercise, but it provides information that is less easily reported than the type, duration, and frequency of a formalized exercise program. Although the LSA was initially developed for the geriatric population, our previous study and the association of the LSA with pedometer counts in this study validate it for the CF population as well. Insofar as measuring physical activity for the purpose of this study, it may be argued that accelerometers are preferable to pedometers. However, we believe this difference is insignificant in this study because the LSA measures only mobility, which is comparable to number of steps by pedometer. Unlike pedometers, accelerometers are able to measure the vigor of the activity, but this is not a parameter assessed by the LSA and would not contribute substantially to our analysis. Furthermore, it has also been shown in community-dwelling older adults that step counts measured by pedometers and accelerometers are closely correlated [34]. Future studies should pilot the use of the LSA questionnaire in regular clinical practice. They should also evaluate it for use in children with CF.

## 5. Conclusion

The Life-Space Assessment score was associated with increased mobility, as assessed by pedometers, and higher lung function in nonhospitalized adults with CF. Future investigation is warranted to determine if the LSA tool can be used to examine the impact of mobility on long-term health outcomes in the CF population.

## Figures and Tables

**Table 1:** Demographics of adult subjects with cystic fibrosis (*N* = 27).

Age (years)	32.15 ± 12.27
Race	
White	26 (96%)
Black	1 (4%)
Sex	
Male	13 (48%)
Female	14 (52%)
FEV_1_% predicted	77.48 ± 22.60
Severity of lung disease by FEV_1_%	
Very severe (<35%)	2 (7%)
Moderate to severe (35%–69%)	8 (30%)
Mild (>69%)	17 (63%)
BMI (kg/m^2^)	23.46 ± 3.76
Mutation	
ΔF508 homozygous	12 (44%)
ΔF508 heterozygous	14 (52%)
Others	1 (4%)
LSA score at enrollment	90.39 ± 22.98
LSA score during study period	91.94 ± 20.64
Pedometer steps at enrollment	19452 ± 10118
Pedometer steps during study period	20213 ± 11331

Reported as mean ± SD or *n* (%).

## Abbreviations

CF: Cystic fibrosis
CFQR: Cystic Fibrosis Questionnaire-Revised
CFQoL: Cystic Fibrosis Quality of Life
FEV_1_% predicted: Forced expiratory volume in one second, percentage of predicted value
HPA: Habitual physical activity
LSA: Life-Space Assessment.
